# Drinking Patterns and Drinking Problems Among Asian-Americans and Pacific Islanders

**Published:** 1998

**Authors:** Kiyoko Makimoto

**Affiliations:** Kiyoko Makimoto, Ph.D., is a professor in the Department of Nursing, Kanazawa University, Japan

**Keywords:** AOD use pattern, problematic AOD use, Asian American, adolescent, college student, adult, prevalence, East Asia, Southeast Asia, South central Asia, socioeconomic status, educational level, acculturation, protective factors, AODE (alcohol and other drug effects), hereditary factors, environmental factors, immigrant, literature review

## Abstract

Researchers are increasingly investigating the drinking behavior and associated consequences among Asian-Americans and Pacific Islanders (APIs) in the United States. Among adolescents, APIs generally have lower rates of alcohol consumption and appear to be at lower risk for alcohol abuse compared with other ethnic groups. Similarly, the rates of drinking and heavy drinking have been found to be lower among API college students than among other ethnic groups. Among adult Asian-Americans, Japanese-Americans have the highest and Chinese-Americans have the lowest lifetime prevalence of drinking and heavy drinking. Southeast Asians (e.g., Vietnamese) living in the United States appear to be at high risk for heavy drinking. Numerous factors help determine the drinking patterns of APIs, including their economic status, educational attainment, and degree of acculturation as well as genetic and environmental factors, such as drinking norms and alcohol availability.

In the past few decades, people of Asian and Pacific Islander (API) ethnicity have been the fastest growing minority in the United States, with their numbers almost doubling between 1980 and 1990 (reviewed in Varma and Siris 1996; Kuramoto 1994). Interest in this population’s alcohol use and abuse also has grown in recent years. The analysis of the drinking practices and alcohol-related medical and social problems of APIs, however, is complicated by that population’s extensive variability (i.e., heterogeneity). Thus, APIs comprise a variety of groups with distinct ethnic and cultural backgrounds. In addition, the immigration history and level of adjustment to the American mainstream culture (i.e., acculturation) vary greatly among, and even within, these ethnic groups. All of these factors may influence drinking behavior among APIs. This article provides a framework for reviewing alcohol use and alcohol problems among APIs in the United States by first summarizing some sociodemographic information. It then reviews studies of the drinking practices of various API subgroups. In addition, the article briefly discusses factors that may cause drinking problems as well as health consequences that may result from alcohol use that are characteristic of the API population.

## Sociodemographic Characteristics of APIs

Although many surveys treat APIs as a single ethnic group, this population is in fact ethnically highly diverse. The 1990 census identified 30 Asian and 21 Pacific Islander ethnic groups (Bennett 1995). Asian-Americans include people of Chinese, Japanese, Indian (e.g., Pakistani, Indian, and Sri Lankan), Korean, Filipino, and Southeast Asian (e.g., Vietnamese, Laotian, Cambodian, Malaysian, and Thai) origin. Pacific Islanders include Polynesians (e.g., Hawaiians, Samoans, and Tongans), Micronesians (e.g., Chamorros), and Melanesians (e.g., Fijians). Even within each of these groups, various subgroups may exist. For example, Chinese-Americans and their ancestors may have come to the United States from Mainland China, Taiwan, or Hong Kong. Among the Laotians, the Hmong, who live in the mountains of northern Laos, form a distinct ethnic group.

Among the APIs in the United States, alcohol use has been studied most frequently in Chinese and Japanese people, followed by Koreans and Filipinos. Southeast Asians generally have been studied separately because their drinking problems tend to differ from those of other Asian-Americans. Little attention has been paid, however, to the drinking practices of Pacific Islanders, at least in part because of the small sizes of many of these groups.

### Differences in Educational Achievement

Of various sociodemographic characteristics studied, both educational attainment and economic status strongly influence drinking patterns and, consequently, risk for alcohol-related problems in the general U.S. population. Accordingly, these characteristics also may influence alcohol use patterns and help explain differences in drinking behavior among APIs. The 1990 census revealed substantial differences in educational achievement among APIs (Bennett 1995). For example, the proportion of Asian-Americans age 25 and older who had completed high school ranged from 31 percent among the Hmong to 88 percent among the Japanese. Among Pacific Islanders, between 64 percent (among Tongans) and 80 percent (among Hawaiians) of adults age 25 and older had a high school diploma. Similar variability existed for the proportion of adults holding a bachelor’s degree: Whereas 58 percent of Indians had obtained at least a bachelor’s degree, only 6 percent or less of Tongans, Cambodians, Laotians, and Hmong had attained that level of educational achievement. These data indicate that both Chinese- and Japanese-Americans, many of whom have been in the United States for several generations, tend to have higher educational achievement than do Caucasians, among whom approximately 85 percent have at least a high school diploma and 24 percent have at least a bachelor’s degree. Conversely, Southeast Asians and Pacific Islanders, many of whom are recent immigrants, tend to gain lower educational achievement than do Caucasians.

### Economic Status

Great heterogeneity also exists in the economic status of APIs. For example, although APIs as a group had a higher median income than Caucasians in 1993 (i.e., $49,510 versus $45,240 per two-parent family), the poverty rate among API families also was higher than among Caucasian families (i.e., 14 percent versus 8 percent) (Bennett 1995). These observations suggest that a bimodal income distribution exists among APIs—that is, some groups of APIs have an above-average economic status, whereas other groups of APIs have a below-average economic status. For example, Japanese, Filipinos, and Indians have much higher median family incomes and much lower poverty rates than do Southeast Asians and Pacific Islanders.

The socioeconomic disparity among APIs reflects differences among, and even within, ethnic groups in both educational preparation (e.g., command of the English language and work experience appropriate for the U.S. job market) and length of stay in the United States. Thus, within various ethnic groups (e.g., Southeast Asians), poverty rates are higher among families with a shorter length of stay in this country than among families with a longer length of stay (Xenos et al. 1987). For some ethnic groups, this association between length of stay and economic status may result from the fact that people who have been in the United States for a longer period of time have had more time to settle and find jobs (Xenos et al. 1987). For other ethnic groups, however, educational and cultural differences exist between immigrants who entered the United States at different times. For example, Southeast Asian refugees who came to this country immediately after the Vietnam War were generally from urban areas and belonged to the elite classes. Conversely, refugees who arrived in the United States during the 1980s were primarily from rural areas and had obtained only minimal education, making their entry into the American workforce more difficult (D’Avanzo 1997).

## Drinking Patterns and Alcohol-Related Problems Among APIs

Several surveys have assessed alcohol consumption and its associated problems among various age and ethnic groups of APIs. Most of these surveys were conducted in the 1980s and involved participants recruited primarily in California, Hawaii, and New York, where most APIs reside.

### Alcohol Consumption Among Adolescent APIs

The drinking patterns of adolescents, including those of API heritage, were investigated in several surveys. For example, in a study conducted in New York City, 27,000 junior and senior high school students were asked about their alcohol and other drug (AOD) use (reviewed in Zane and Kim 1994). Two percent of the students were of API descent. Among the various ethnic groups included in the study, API students reported the lowest percentage of alcohol use in the past year. Furthermore, the percentage of heavy drinkers (i.e., students who drank at least once per week and consumed five or more drinks^1^ per occasion) was substantially lower among API students than among Caucasian students (i.e., 6 percent versus 16 percent). Among the heavy drinkers, however, API students reported greater alcohol consumption per day than did their Caucasian counterparts (i.e., 1.46 ounces versus 0.76 ounces). Other studies conducted in California also found that API students had lower rates of alcohol use than did other ethnic groups (Zane and Kim 1994). These and all other published surveys treated API adolescents as one racial group and did not examine differences in drinking practices among different ethnic groups of APIs.

Studies such as the ones described here may not present an accurate picture of alcohol consumption among API adolescents. For example, school-based surveys may not include students at high risk for heavy alcohol consumption, because those students often drop out of school. Furthermore, API families rarely seek treatment for alcohol problems, because they want to avoid the shame and disgrace traditionally associated with admitting these problems outside the family (James et al. 1997). Both factors may result in an underrepresentation of API adolescents with alcohol problems.

In an attempt to identify some of the reasons underlying AOD use among API adolescents, James and colleagues (1997) studied 21 Asian students in grades 8 to 12 who had been referred for an assessment of suspected AOD use in Seattle, Washington. Of these students, 14 percent were diagnosed with AOD misuse (i.e., AOD use resulting in intoxication and impairment). Another 24 percent were diagnosed with abuse (i.e., AOD use that was continual, occurred episodically or in binges, or was in remission), and 24 percent were diagnosed with chemical dependency (i.e., physical dependence on AODs). The researchers suggested that the students’ AOD use problems were primarily caused by stresses related to the transition to Western culture, which disrupted the APIs’ hierarchical family structure, their interdependence with other family members, and the self-identity of young APIs (James et al. 1997).

When analyzed together, the studies reviewed in this section indicate that API adolescents appear to be at a low risk for alcohol abuse, although certain high-risk groups (e.g., school dropouts) do exist in this population. For more accurate information, school-based alcohol use surveys should be supplemented by information on treatment referral and treatment use of API adolescents as well as through surveys of key informants (e.g., parents and teachers).

### Alcohol Use Among API College Students

Drinking patterns among APIs also have been investigated using college student samples. Across surveys, both the percentage of drinkers and the percentage of heavy drinkers invariably were lower among API students than among Caucasian students (Zane and Kim 1994). These findings are consistent with surveys of the drinking behavior of APIs in the general population.

One study also assessed the influence of the degree of acculturation on the students’ drinking behavior. The level of acculturation was determined from the number of generations the student’s family had been in the United States and from the student’s ability to speak the ethnic language. In that study, the more strongly acculturated API students reported higher levels of alcohol consumption than did the less strongly acculturated students (reviewed in Zane and Kim 1994). This observation suggests that with increasing acculturation, APIs also adopt the drinking norms of the mainstream culture.

The college-based studies also have limitations, however, with respect to their ability to evaluate drinking patterns of all APIs, including those at high risk for heavy drinking. For example, most survey participants were recruited among students at one particular university. Consequently, the participants may not have represented college-age APIs in general. In addition, the college students by definition were more acculturated; consequently, their drinking patterns may not represent those of APIs who have only limited language or job skills or who have recently immigrated to the United States. Finally, the small sample sizes of all surveys reviewed in this article allowed no comparisons of the drinking patterns among ethnic groups of APIs.

### Drinking Patterns of Adult Chinese-, Japanese-, Korean-, and Filipino-Americans

Numerous studies have compared alcohol use patterns among various ethnic groups of Asian Americans, particularly Chinese-, Japanese-, Korean-, and Filipino-Americans. The definitions of drinking and heavy drinking used in the studies vary, however, making it difficult to compare drinking levels across studies. Nevertheless, surveys conducted in California and Hawaii frequently found that Japanese-Americans had the highest, and Chinese-Americans had the lowest, percentage of both lifetime drinkers and heavy drinkers (see Parrish 1995; Varma and Siris 1996; Zane and Kim 1994). For example, in one large survey among APIs in California, 69 percent of Japanese-Americans, 49 percent of Korean-Americans, 38 percent of Filipino-Americans, 36 percent of Vietnamese-Americans, and 25 percent of Chinese-Americans reported consuming 10 or more drinks in their lifetime (reviewed in Zane and Kim 1994). These rates are all substantially lower than the lifetime alcohol use rate of 85 percent among the general U.S. population. Other studies found, however, that although Korean-Americans had the highest rates of abstainers among various API groups, Korean-Americans had the highest rates of heavy drinkers among those who did consume alcohol (see Parrish 1995).

The drinking patterns of APIs are determined not only by their ethnicity but also by their place of birth. An analysis of the drinking levels of APIs enrolled in a health maintenance organization demonstrated that the percentage of abstainers was lower among APIs born in the United States than among those born outside the United States (see Parrish 1995). Similarly, in a survey conducted in Hawaii, the Chinese-, Filipino-, and Japanese-Americans who were born in Hawaii had substantially lower abstention rates than did those who had been born outside the United States (see Parrish 1995).

Little is known about the alcohol consumption patterns and alcohol-related problems of Indians. This lack of information is probably attributable to the short immigration history and relatively small number of Indian immigrants. As mentioned earlier in this article, Indians have the highest proportion of people with advanced academic degrees among API immigrants. Because studies in the general U.S. population have indicated that educational attainment is related to alcohol use patterns (National Institute on Alcohol Abuse and Alcoholism 1997), this observation suggests that the drinking practices of Indians may differ from those of other APIs.

Not much is known about the utilization and effectiveness of alcoholism treatment among Chinese-, Japanese-, Korean-, and Filipino-Americans. These ethnic groups generally are underrepresented in AOD treatment centers (i.e., their proportion in the treatment population is lower than in the general population) (Zane and Kim 1994). Similarly, hospital admission rates for alcohol-related problems are low among these Asian-American groups. These observations do not necessarily mean, however, that Asian-Americans have fewer alcohol-related problems than do other ethnic groups; rather, they may reflect a reluctance of this population to use existing services. In fact, some studies have demonstrated that treatment utilization by Asian-Americans increases substantially if bilingual and bicultural personnel provide the treatment services (Zane and Kim 1994).

### Drinking Practices of Southeast Asians

As previously discussed, Southeast Asians, particularly Vietnamese and Cambodians, emigrated to the United States in two waves, each of which comprised people of vastly different educational and cultural backgrounds. The first wave, which arrived shortly after the Vietnam war in 1975, included mostly well-educated people who had lived in urban areas. Conversely, immigrants who arrived with the second wave, during the 1980s, frequently had been peasants living at a subsistence level. Most of these immigrants were illiterate in their native language as well as in English and were ill equipped for life in the United States (D’Avanzo 1997). In addition, many of them had experienced several years of political unrest, imprisonment, severe physical and psychological violence, starvation, and long stays in refugee camps. As a result of these experiences, psychological disorders, particularly depression and posttraumatic stress disorder (PTSD), are common among this population (Amodeo et al. 1997).^2^

Small-scale studies have indicated that Southeast Asians living in the United States are at high risk for heavy alcohol consumption. For example, a study of Vietnamese living in California demonstrated that although the percentage of drinkers was similar among Vietnamese males and among males in the general American population, binge drinking was twice as common among Vietnamese males as among non-Southeast Asian males (see Amodeo et al. 1997). Several factors may contribute to the high risk of alcohol problems among Southeast Asians (Amodeo et al. 1997):

- Many Southeast Asian cultures do not consider alcohol a harmful drug but, rather, a substance with healing properties.
- As mentioned previously, many Southeast Asian immigrants suffer from depression, PTSD, and other psychological problems. They may use alcohol as self-medication to provide temporary relief from their symptoms and from memories of traumatic experiences.
- Alcohol is more readily available in the United States than in the Southeast Asian countries.
- Many Southeast Asians living in the United States are children of American soldiers and Southeast Asian mothers who came to this country to locate their fathers. Many of these people did not find or were rejected by their American families; consequently, they often feel marginalized in American society.

The role of these factors in contributing to alcohol problems is supported by findings from studies that evaluated Southeast Asians who were undergoing alcoholism treatment. For example, in an investigation at the addiction treatment center of the University of Minnesota Hospital and Clinic, 38 of 50 Southeast Asians who sought treatment reported that they used AODs specifically to relax and enjoy life (D’Avanzo 1997). Similarly, a study of youth of various ethnicities found that Southeast Asian youth who had recently immigrated to the United States drank primarily to forget their pasts, whereas members of other ethnic groups drank mostly for social reasons (Morgan et al. 1984). Accordingly, Southeast Asians with psychiatric problems should be evaluated for AOD problems (Amodeo et al. 1997).

### Drinking Practices of Pacific Islanders

To date, the alcohol consumption patterns of Pacific Islanders have not been examined in detail. The available studies generally have focused on native Hawaiians, comparing them with other ethnic groups living in Hawaii. For example, one study has suggested that Hawaiians have more serious AOD problems than do members of other major ethnic groups (see Mokuau 1996). In that study, Hawaiians had the highest rates of binge drinking (i.e., 5 or more drinks per occasion at least once per month) and chronic drinking (i.e., 60 or more drinks during the past month) compared with Caucasians, Filipinos, and Japanese living in Hawaii. Other surveys conducted in Hawaii also demonstrated that alcohol consumption was higher among Hawaiians than among Japanese, Chinese, and Filipinos (see Mokuau 1996). The drinking practices of other groups of Pacific Islanders have not been explored in detail, at least in part because many of these groups are small.

## Determinants and Health Effects of Drinking Among APIs

Not much is known about the determinants of drinking among APIs, especially because these determinants may differ substantially among, and even within, ethnic groups. Moreover, in many studies the sample sizes are too small to control for major demographic variables (e.g., age and socioeconomic status). Various studies, however, have focused on the influence of acculturation on drinking behavior. For example, Kitano and colleagues (1992) compared drinking norms and alcohol consumption among Japanese living in Japan, Hawaii, and California. The study demonstrated that both attitudes about drinking and drinking behavior largely reflected the attitudes and behavior of the mainstream culture in which the respondents were living. Other studies also found that acculturation at least partly explains the observed differences in drinking levels among APIs (e.g., Tsunoda et al. 1992). Thus, the drinking practices of APIs born in the United States were more likely to reflect the predominant attitudes of U.S. culture than the cultural attitudes of their ancestors.

When evaluating and interpreting the findings of these studies, however, it is important to keep in mind that factors other than acculturation may contribute to the observed differences in drinking behavior, including the following^3^ (Parrish 1995):

- Both in the United States and in some Asian countries, drinking behaviors have changed substantially over the past few decades. These changes may confound the effect of acculturation.
- The socioeconomic status of the descendants of API immigrants has changed over the years (e.g., educational attainment and economic status may have improved). These factors may influence drinking behavior independently of the degree of acculturation.
- APIs who immigrated to the United States were not representative of the general population of their homelands. Accordingly, the drinking practices of the immigrants may have already differed from those of the general population in those countries.
- Demographic variables (e.g., socioeconomic status, religious affiliation, and marital status) of the API immigrants and their descendants may differ from those of other ethnic groups (e.g., African-Americans and Hispanics) in the United States. These differences also may account for differences in drinking behavior and confound the influence of acculturation.

### Protective Factors

In recent years, researchers have suggested that genetic factors may contribute to the lower rates of alcohol use and alcoholism observed among APIs. Alcohol is broken down in the body by two enzymes: (1) alcohol dehydrogenase, which converts alcohol to acetaldehyde, and (2) aldehyde dehydrogenase (ALDH), which converts acetaldehyde to acetate. ALDH inactivity results in the accumulation of acetaldehyde in the body, which leads to the so-called flushing reaction. This reaction is characterized by numerous symptoms, such as facial flushing, nausea, headache, dizziness, and rapid heartbeat. Several genes code for the ALDH enzyme, including the ALDH2 gene. Two variants of the ALDH2 gene exist. The ALDH2^1^ variant produces a functional enzyme, whereas the ALDH2^2^ variant produces an inactive enzyme. Each person inherits two copies of the ALDH2 gene, one from the father and one from the mother. People who inherit two copies of the ALDH2^2^ gene or one copy of the ALDH2^1^ gene and one copy of the ALDH2^2^ gene produce an impaired ALDH enzyme and are susceptible to the flushing reaction (Wall et al. 1996). Because they experience the unpleasant flushing reaction, these people may consume less or no alcohol and therefore be at reduced risk for alcoholism.

The inactive ALDH2^2^ gene variant is not equally common among all ethnic groups. Few Caucasians but up to 50 percent of Asians carry one or two copies of the ALDH2^2^ gene and are therefore susceptible to flushing (Wall and Ehlers 1995). Several studies have found substantial differences in the rates of alcohol consumption and alcoholism among people with no, one, or two copies of the ALDH2^2^ gene. For example, some studies found that Asians who carried two copies of the ALDH2^2^ gene drank little alcohol and were not found among groups of alcoholics (see Wall and Ehlers 1995). Furthermore, Asians who carried one copy of the ALDH2^2^ gene drank significantly less and had significantly lower rates of alcoholism compared with Asians who carried two copies of the functional ALDH2^1^ gene (Wall and Ehlers 1995). These findings indicate that the presence of an inactive ALDH2^2^ gene may protect Asians, at least partially, against heavy drinking and the risk of alcoholism.

In addition to genetic influences, however, environmental influences probably also play an important role in determining the drinking behavior of APIs. For example, cultural norms that emphasize moderate drinking or abstinence may result in lower alcohol consumption and thus a reduced risk of alcoholism in Asian populations. Accordingly, changes in drinking norms that are more permissive toward alcohol consumption should lead to increased consumption levels. Such changes have been observed in Japan, where attitudes have become more tolerant toward drinking, particularly among women, since World War II. As a result of those changes, Japan has experienced a fourfold increase in per capita alcohol consumption over the past four decades. Moreover, the increase in the percentage of drinkers has been more pronounced in young women than in any other group (Parrish et al. 1991). These observations suggest that drinking norms and the availability of alcoholic beverages probably have at least as great an impact on alcohol consumption in Asian populations as do genetic factors.

### Health Effects of Alcohol Abuse

Alcohol abuse adversely affects many organ systems and has numerous health consequences. To determine the health effects of heavy alcohol consumption among the entire population, however, researchers frequently monitor cirrhosis mortality. Cirrhosis was chosen because heavy drinking is by far the most important risk factor for that disorder.^4^ Even the analysis of cirrhosis mortality, however, is problematic when used for ethnic minorities, because the number of deaths from cirrhosis in these groups generally is too small to establish valid mortality rates. In one of the few studies conducted to date, Gardner (1994) compared cause-specific mortality rates among APIs and Caucasians. For the time period of 1979 and 1980, the analysis determined age-adjusted cirrhosis mortality rates of 6.9 per 100,000 for Asian-American males and 15.4 per 100,000 for Caucasian males. These numbers confirm that heavy drinking is less prevalent among Asian-Americans than among Caucasians. The study did not examine, however, whether differences in cirrhosis mortality existed among various ethnic groups of Asian-Americans.

The use of cirrhosis mortality as an indicator of alcoholism is confounded, however, by the presence of viral hepatitis infections, which also cause cirrhosis. The prevalence of viral hepatitis may differ among ethnic and age groups. For example, in a 1980 age-specific comparison of cirrhosis mortality rates between whites in the United States and Japanese, among subjects between ages 35 and 64, Japanese men had the highest cirrhosis mortality rates, followed by white men, white women, and Japanese women. Conversely, in older age groups (i.e., ages 65 and over), Japanese women had the second highest mortality rates, followed by white men and white women. Because the average alcohol consumption among the Japanese women was very low, the differences in cirrhosis mortality rates reflect a higher prevalence of viral hepatitis infections in Japanese than in whites in the United States (Parrish et al. 1991) and suggest that APIs may have a higher prevalence of viral hepatitis. Therefore, researchers must take into consideration the prevalence of viral hepatitis infections when comparing cirrhosis mortality rates among different ethnic groups.

## Summary

APIs, particularly Asian-Americans, are the fastest growing ethnic minority in the United States. Moreover, the demographic profile of the Asian-American population is changing rapidly. Filipino-Americans now outnumber Chinese-Americans, who once were the largest Asian-American group. Likewise, the Vietnamese-American population is expected to exceed the Chinese-American population by the year 2000. Other Southeast Asian populations also will likely continue to grow. However, the drinking patterns and consequences of alcohol use in these populations have not been studied adequately in recent years. Future studies must address the determinants and characteristics of alcohol use among Asian-Americans in order to develop and provide adequate treatment services to these populations. Such studies also must consider the heterogeneity of the Asian-American population as well as the cultural differences within ethnic groups. Additional studies also should focus on Pacific Islanders, because little information is currently available regarding their drinking practices.

## Footnotes

1 A standard drink is defined as 12 ounces of beer or wine cooler, 5 ounces of wine, or 1.5 ounces of distilled spirits, each of which contains approximately 0.5 ounce of pure alcohol.

2 It is currently unknown whether substantial differences in drinking behavior and alcohol-related problems exist between those groups, but one can presume that second-wave immigrants may have more substantial alcohol-related problems than first-wave immigrants.

3 The exact contributions of these factors to drinking behavior, as well as the extent to which these factors are truly independent from acculturation processes, may be difficult to determine.

4 Other alcohol-related health consequences, such as esophageal and liver cancer, also are associated with various other risk factors that may differ among ethnic groups.
